# Algorithm-based quantification of tissue vascularization in immunohistochemical stainings of tissue sections

**DOI:** 10.1038/s41598-026-56867-x

**Published:** 2026-06-08

**Authors:** Ario Dastmaltschi, Tarik Bozoglu, Vijayanand Rajendran, Seyed Amir Shakouri, Shriyam Bhardwaz, Denise Messerer, Simone Renner, Andrea Bähr, Eckhard Wolf, Tobias Weinberger, Hendrik B. Sager, Karl-Ludwig Laugwitz, Christian Kupatt, Tilman Ziegler

**Affiliations:** 1 Clinic and Polyclinic for Internal Medicine I, TUM University Hospital Rechts der Isar, Munich, Germany; 2 Department of Cardiology, TUM University Hospital German Heart Center Munich, Munich, Germany; 3 Medical Clinic I, Department of Cardiology, LMU Klinikum, Munich, Germany; 4 Center for Innovative Medical Models (CiMM) of the veterinary faculty, LMU Munich, München, Germany; 5 DZHK (German Centre for Cardiovascular Research), Partner Site Munich Heart Alliance, Munich, Germany; 6 TUM University Hospital Rechts der Isar, Ismaninger Str. 22, 81675 München, Germany

**Keywords:** Biological techniques, Cell biology, Computational biology and bioinformatics

## Abstract

The vascular system ensures sufficient blood supply and tissue homeostasis and consists of different cell types. Endothelial cells represent the structural backbone of blood vessels and are accompanied by mural cells, specifically pericytes in the microcirculation as well as vascular smooth muscle cells (vSMC) along the larger vessels (arteries, arterioles and veins). Distinguishing these different cell types in immunohistochemical stainings presents a challenge due to their close proximity and the unreliable marker distribution of mural cells. Furthermore, manual quantification of capillaries and pericytes is highly examiner-dependent, hindering inter-examiner and inter-laboratory comparisons. To address these issues, we developed an automated algorithm-based analysis software designed to standardize quantification of vascular structures in immunohistochemical images, named CAPPER (Capillary / Pericyte Quantification Tool). Through the implementation of adaptive thresholding, morphological operations, and domain-specific knowledge, CAPPER excels in the quantification of capillary densities and pericyte coverage in different organs (brain, heart, muscle, and kidney), species (mouse and pig) as well as a plethora of disease states. We furthermore propose a robust pericyte marker array to more accurately identify this elusive cell type.

**Supplementary Information:**

The online version contains supplementary material available at 10.1038/s41598-026-56867-x.

## Introduction

The vascular system is a hierarchical network of vessels essential for delivering oxygen and nutrients, removing waste products, and maintaining tissue homeostasis. Structurally, it consists of endothelial cells that form the vessel lumen and mural cells that provide support and regulate vascular tone. Depending on the vessel size, mural cells are either vascular smooth muscle cells (vSMCs), which surround arteries, arterioles, and veins, or pericytes (PCs), which are closely associated with capillaries^1–3^. Pericytes are embedded within the vascular basement membrane and are critical for regulating endothelial stability, vascular permeability, and angiogenesis^1–3^.

Despite their importance, reliable identification of pericytes remains a longstanding challenge in vascular biology. Unlike endothelial cells, which are uniformly marked by proteins such as CD31 (PECAM-1), pericytes display a heterogeneous expression pattern across tissues and under different physiological or pathological states. Commonly used pericyte markers are NG2 (Neural/glial antigen 2) and PDGFR-β (Platelet-derived growth factor receptor β), but no universally specific or consistently expressed marker has been identified to date. This heterogeneity complicates interpretation of immunohistochemical studies and leads to discrepancies in reported pericyte coverage between studies.

Another obstacle in vascular analysis lies in the quantification process. Manual counting of capillaries and pericyte associations is labor-intensive and prone to user bias. Manual methods are therefore limited in throughput and reproducibility, hampering comparative studies across tissues, experiments, and laboratories.

Automated image analysis has emerged as a powerful tool to standardize quantification and reduce human error in histology. Several dedicated workflows for automated analysis of capillaries and/or pericytes have been described, including QuPath-based whole-brain section pipelines^4^, stereological and two-photon-based approaches for capillary and pericyte quantification in the central nervous system^5^, and general-purpose vascular analysis tools for brain and retina^6–8^. However, the majority of these approaches are tailored to a single tissue type, rely on transgenic reporter lines, or are limited to capillary density without integrated per-capillary pericyte coverage, and a broadly applicable pipeline that combines capillary density, pericyte coverage and marker specificity across multiple organs and species is still lacking.

In this study, we present a novel algorithm named CAPPER (**Cap**illary **/ Per**icyte Quantification Tool) developed in Python that integrates adaptive thresholding, morphological filtering, and domain-specific knowledge to automatically quantify capillary density and pericyte coverage. CAPPER enables comparative evaluation of pericyte markers, addressing the long-standing issue of marker variability. We validated the tool across four different organs (heart, brain, muscle, and kidney) in mice and pigs as well as different disease states and demonstrated its ability to resolve biologically meaningful differences in vascular composition. By minimizing examiner-dependence and improving reproducibility, this approach provides a significant advance for vascular biology and offers a scalable framework for histological quantification.

## Results

### Algorithm

**Fig. 1:**
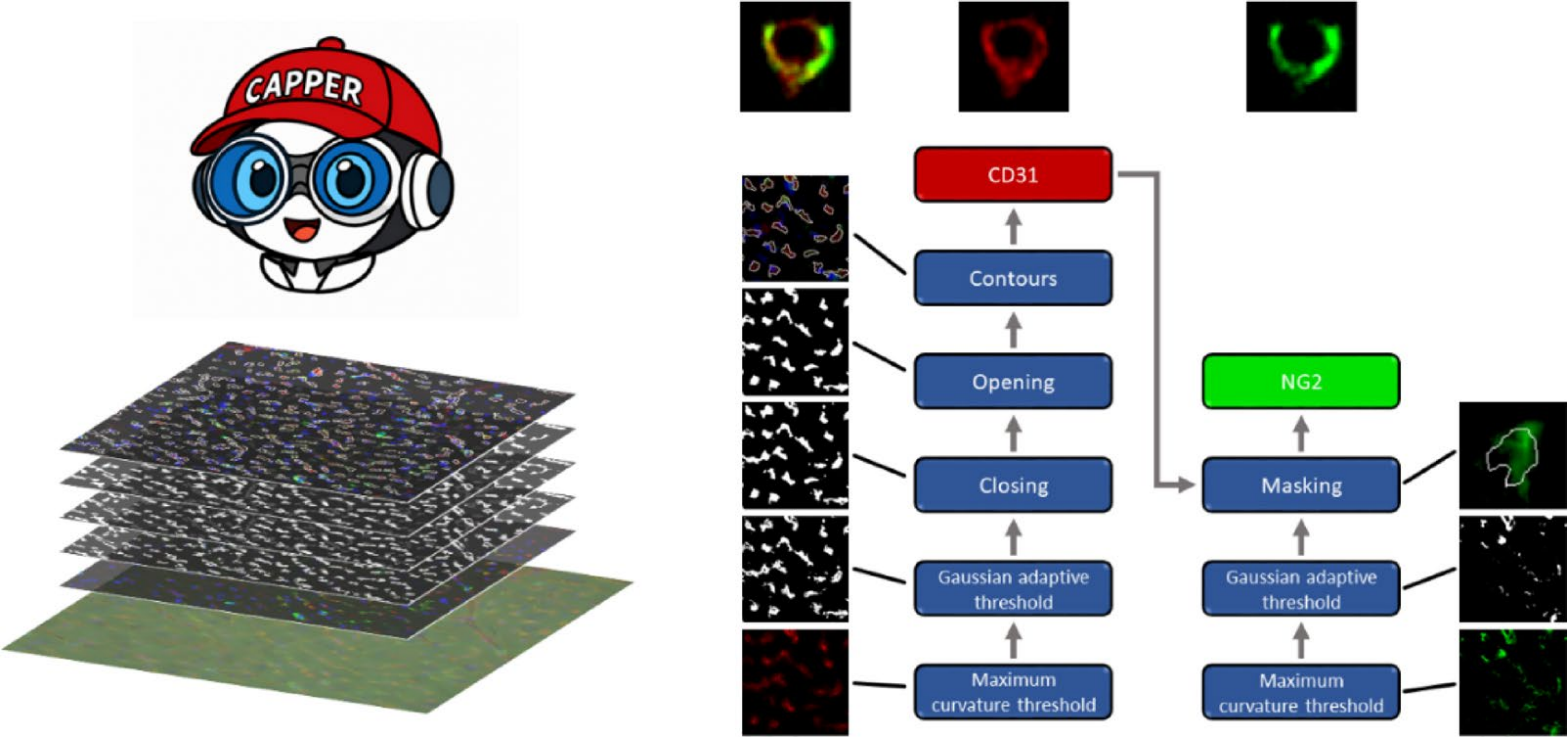
schematic representation of the CAPPER workflow. The CD31 signals of raw images were optimized for automatic counting and contouring using blurring and subsequent Gaussian adaptive thresholding, closing and opening. Similarly, NG2 positive signals underwent blurring and Gaussian adaptive thresholding followed by the overlay of the CD31 signal to quantify NG2 coverage of CD31 signals.

We developed a reproducible workflow for automated vascular analysis by combining adaptive thresholding and morphological filtering to process large-scale immunohistochemical images. The workflow is visualized in Fig. 1. In short, endothelial cells were identified using PECAM-1 (CD31), pericytes were stained with NG2, a widely used marker of this cell type. 16-bit images were separated into the respective channels and processed in 500 × 500 pixel tiles to correct for staining variability and artifacts. Thresholding followed the method of Klauschen et al.^9^, using histogram-based maximum curvature estimation. CD31-positive endothelial structures were finally segmented with Gaussian blurring, adaptive thresholding, and morphological operations, then filtered by size to isolate true capillaries. Pericyte coverage was quantified by applying a CD31-derived mask to NG2 signals within each capillary contour and calculating the NG2-to-CD31 ratio. This optimized pipeline enables rapid processing, completing an 8000 × 8000 pixel image in seconds on standard hardware even without parallel processing capabilities from GPUs (for a more detailed description of the algorithm see also Methods).

### Organ specific quantification of microcirculatory structures

**Fig. 2:**
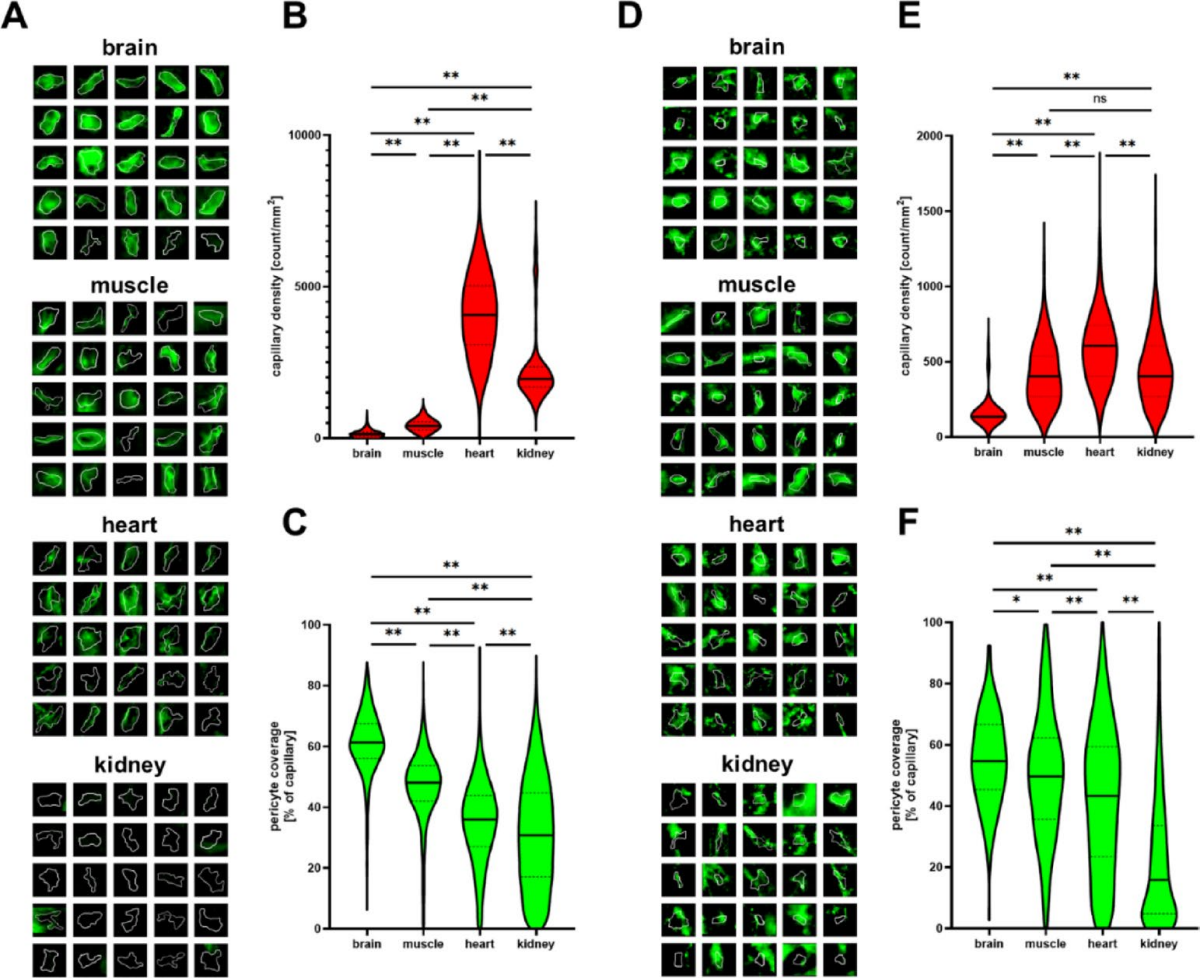
(**A**) example images from different organs of mice showing NG2 overlaid over CD31 outline, allowing for coverage quantification. (**B**) quantification of capillary density in mice demonstrates low capillarization of the brain in comparison to peripheral muscle, kidney and heart. (**C**) pericyte coverage in different organs with the highest coverage in the brain followed by muscle, heart, and kidneys. (**D**) example images of porcine organs visualizing pericyte overlay alongside (**E**) capillarization and (**F**) pericyte coverage, highlighting the same distribution of capillarization and pericyte coverage as seen in mice (* *p* < 0.05 / ** *p* < 0.001).

To validate the capacity of CAPPER to quantify the capillary density and pericyte coverage of those capillaries, we compared PECAM-1 / NG2 stainings of different organs (brain, heart, peripheral muscle, and kidney) from mice to published data. By outlining the capillary structures – based on the staining for CD31 – our approach is able to quantify both capillary density as well as pericyte coverage (Fig. 2 A). Concerning capillaries, CAPPER calculates capillary density values (in capillaries/mm^2^ or c/mm^2^) similar to published numbers^10–15^ with the highest capillary density in the heart (4045 ± 55 c/mm^2^) followed by the kidneys (2290 ± 58 c/mm^2^), peripheral muscle (437 ± 10 c/mm^2^) and brain (151 ± 6 c/mm^2^, Fig. 2 B).

Due to the difference in capillary density between the organs, different amounts of capillaries per organ were analyzed (Supplementary Fig. 1 A). After automated contouring of the capillaries, NG2 signals within those contours were quantified to calculate the pericyte coverage in the aforementioned organs (Fig. 2 C). The automated analysis of pericyte coverage shows a comparatively high abundance of pericytes in the brain (61 ± 0.3 in % of capillaries) as compared to the other organs – peripheral muscle (47 ± 0.2%), heart (35 ± 0.1%), and kidney respectively (31 ± 0.1%, Fig. 2 C). These findings are in line with previously published data^16–18^. Not only does the amount of pericyte coverage differ between organs, we furthermore demonstrate a high heterogeneity in the pericyte coverage of the kidney capillary surface. In contrast, brain and peripheral muscle tissue sections reveal a narrower variation of pericyte coverage compared to the kidneys, with the heart demonstrating an intermediate pattern (Fig. 2 C, Supplementary Fig. 1 B).

Since CAPPER proved capable in identifying the amount of capillaries per organ as well as the pericyte coverage in mice – a standard animal model in basic research – we aimed to challenge CAPPER with an additional animal species, namely pigs. Particularly in porcine immunohistochemical specimen, the analysis of capillary features is substantially more demanding due to a limited availability of suitable antibodies and staining protocols as well as a more pronounced tissue autofluorescence in pigs. Similar to our previous line of experiments we utilized CAPPER to identify capillary density and pericyte coverage in the respective porcine specimen (brain, heart, peripheral muscle and kidney) after staining for PECAM-1 and NG2.

In line with the results from our mouse study, we could demonstrate a similar distribution of capillary densities with the highest numbers in the heart followed by kidneys, peripheral muscle and lastly the brain (Fig. 2 D + E). Also in pigs, the pericyte coverage of capillaries was highest in the brain with 54.9 ± 1.5 in % of capillaries with lower pericyte coverage in the peripheral muscle (50.3 ± 0.4%), heart (42.2 ± 0.5%) and kidneys (21.8 ± 0.5%), mirroring the results gathered in mice (Fig. 2F).

We thus demonstrate that CAPPER not only reliably quantifies capillary density across different organs but also between relevant experimental species.

### Changes of capillary density and pericyte coverage in disease states

Capillary density as well as pericyte coverage is prone to dramatic changes depending on exercise status as well as disease states. To investigate if CAPPER is capable of detecting changes in these parameters under different circumstances, we analyzed murine peripheral muscle during exercise, the hearts of pigs suffering from the cardiovascular risk factors hypercholesterolemia and diabetes as well as mouse hearts during different cardiac disease states. Lastly, we investigated the effects of a pro-angiogenic gene therapy approach in porcine hindlimb ischemia.

**Fig. 3:**
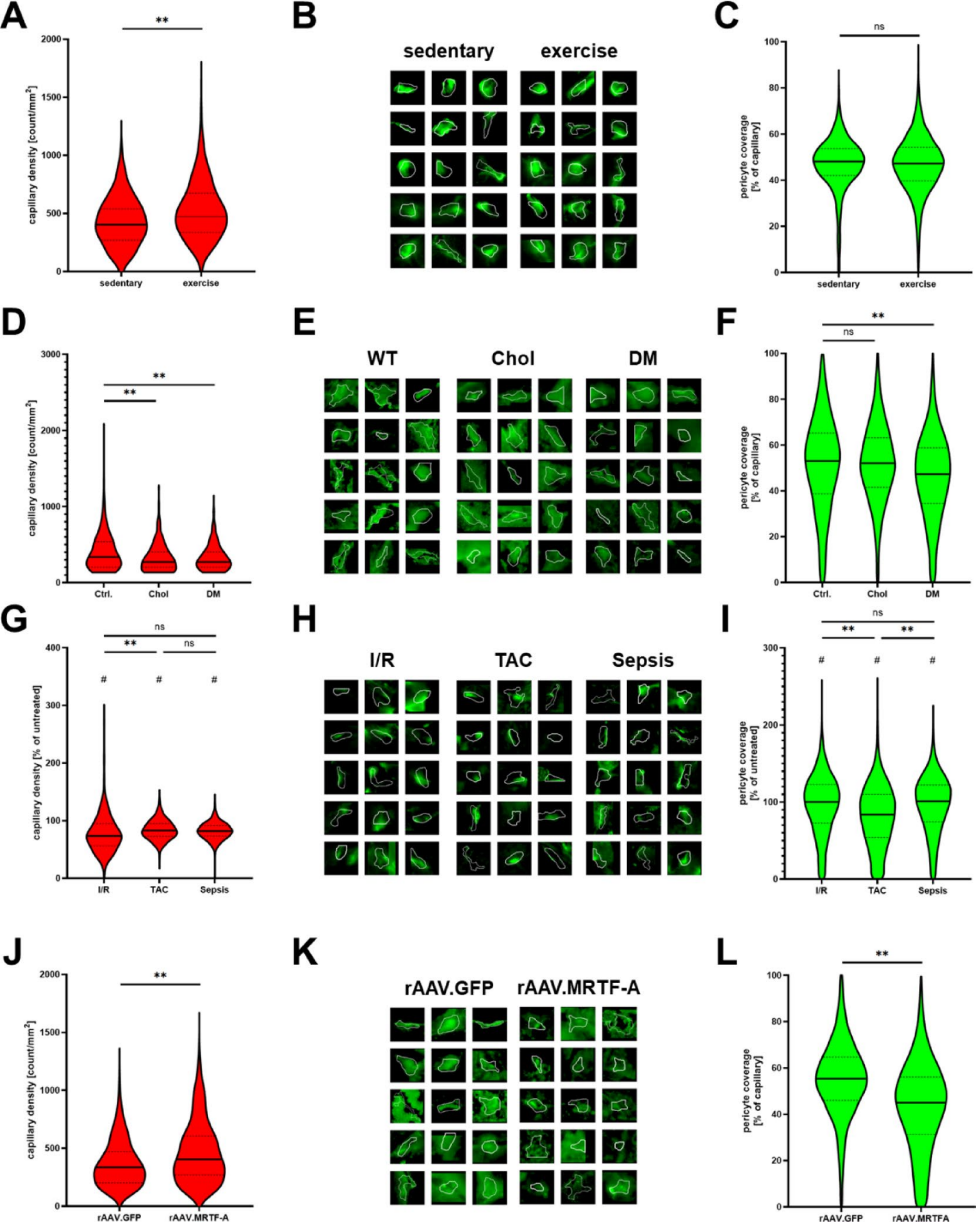
(**A**) Capillary density and (**B** + **C**) pericyte coverage in mice who either exercised or were sedentary demonstrate an increase in capillarization without a change in pericyte coverage. (**D**) capillary density and (**E** + **F**) pericyte coverage in wild-type pigs as well as hypercholesterolemic and diabetic pigs demonstrate a decrease in capillarization as well as pericyte coverage under hypercholesterolemia as well as diabetes to a comparable degree. (**G**) Capillarization and (**H** + **I**) pericyte coverage (both displayed as a percentage of healthy controls) of murine hearts in different disease states shows a decrease in capillary density in all states, most prominently in the ischemic myocardium with a less pronounced decrease in pericyte coverage, while the highest loss in pericyte coverage can be seen during TAC. (**J**) Pigs undergoing chronic hindlimb ischemia reveal an increase in capillarization upon transduction with MRTF-A, while pericyte coverage decreases K + L (* *p* < 0.05 / ** *p* < 0.001 / # *p* < 0.05 vs. Ctrl.)

Considering exercise as a potent stimulus increasing the capillarization of peripheral muscles, mice undergoing exercise (six weeks of voluntary running) show an increase in capillarization of the peripheral muscle (Fig. 3 A) compared to sedentary wild-type mice as expected^19^, while pericyte coverage overall remained unchanged (Fig. 3 B + C) indicating a gain of functional, pericyte-controlled capillary structures.

Since both diabetes as well as hypercholesterolemia are known to induce low-level inflammation resulting in a loss of pericytes^20,21^ and capillarization^22,23^, we furthermore investigated the ability of CAPPER to detect the changes in both parameters in pigs carrying these risk factors. Of note, we could observe a drop in capillarization in hypercholesterolemic and diabetic pigs alike (Fig. 3 D) with an accompanying drop in pericyte coverage (Fig. 3 E + F).

Other widespread clinical challenges for the microvasculature are constituted by ischemia/reperfusion injury, systemic inflammation / sepsis and pressure overload – as a result of aortic stenosis or hypertension, which can be recapitulated via transverse aortic constriction (TAC) in mice. CAPPER, again, was well suited to quantify capillary density and pericyte coverage in these states.

In ischemia/reperfusion injury CAPPER demonstrates the most drastic loss of capillarization (77% of ctrl. Figure 3 G) compared to the other two disease models with a mild drop in pericyte coverage to 96% of ctrl. (Fig. 3 H + I, Supplementary Fig. 2 A + B). The effect of ischemia/reperfusion injury on endothelial dysfunction and loss in pericytes is well documented, caused mostly by direct damage to the endothelial layer via reactive oxygen species, mitochondrial dysfunction and sterile inflammation caused by the sudden influx of oxygenated blood. The near normal pericyte investment of the remaining capillaries speaks to the fact, that endothelial cells are the main structures suffering damage during I/R^24–26^. While the drop in capillaries is less pronounced in pressure overload mediated by transverse aortic constriction (84% of ctrl.), the drop in pericyte coverage (81% of ctrl.) in these mice is the most pronounced with 20% pericyte loss of remaining capillaries. That capillary rarefication is caused by insufficient angiogenic growth signaling relative to cardiomyocyte hypertrophy^27^, while the loss in pericyte coverage can be attributed to altered signaling through the Angiopoietin / Tie2 and PDGF-B / PDGFR-β axes due to chronic mechanical stress^28,29^. In line with data from our group, CAPPER demonstrated a loss in capillaries (82% of ctrl.) as well as pericyte coverage (81% of ctrl.) during LPS induced systemic inflammation, which is mostly caused by a rapid increase in Angiopoietin-2, destabilizing endothelial-pericyte interaction^30^. Indeed, CAPPER was able to confirm the accepted responses of the microcirculatory unit to relevant pathological stimuli while highlighting the differences in capillary structures based on those stimuli.

Lastly, to investigate the performance of CAPPER in a gene-therapeutic setting, particularly providing growth and maturation of capillary structures, we analyzed the microcirculation in pigs undergoing chronic hind-limb ischemia who were – additionally – subjected to an rAAV based gene therapy causing overexpression of MRTF-A, a potent driver of vascular sprouting as well as microvessel stabilization^31–33^. Consistently, CAPPER revealed an increase in capillary density under MRTF-A overexpression in porcine ischemic hindlimbs compared to control transduced animals (rAAV.GFP 370.8 ± 9.4 c/mm^2^ / rAAV.MRTF-A 465.3 ± 10.4 c/mm^2^, Fig. 3 J) while pericyte coverage was diminished (rAAV.GFP 54.9 ± 0.3% / rAAV.MRTF-A 43.4 ± 0.3%, Fig. 3 K + L). Although pericyte coverage was decreased, overall perfusion of the muscle should improve after MRTF-A transduction. Mechanistically, the MRTF-A/SRF axis drives different gene expression outputs depending on the cell type^34^. Endothelial cell restricted overexpression as used here^35^ might either only drive capillary sprouting without recruitment of pericytes or require a longer period to result in pericyte recruitment.

In summary, our data validate established aspects of microcirculatory biology and demonstrate the robustness and scalability of our approach for high-throughput quantification of microvascular composition in diverse experimental settings.

### Evaluation of optimal pericyte marker combinations

**Fig. 4:**
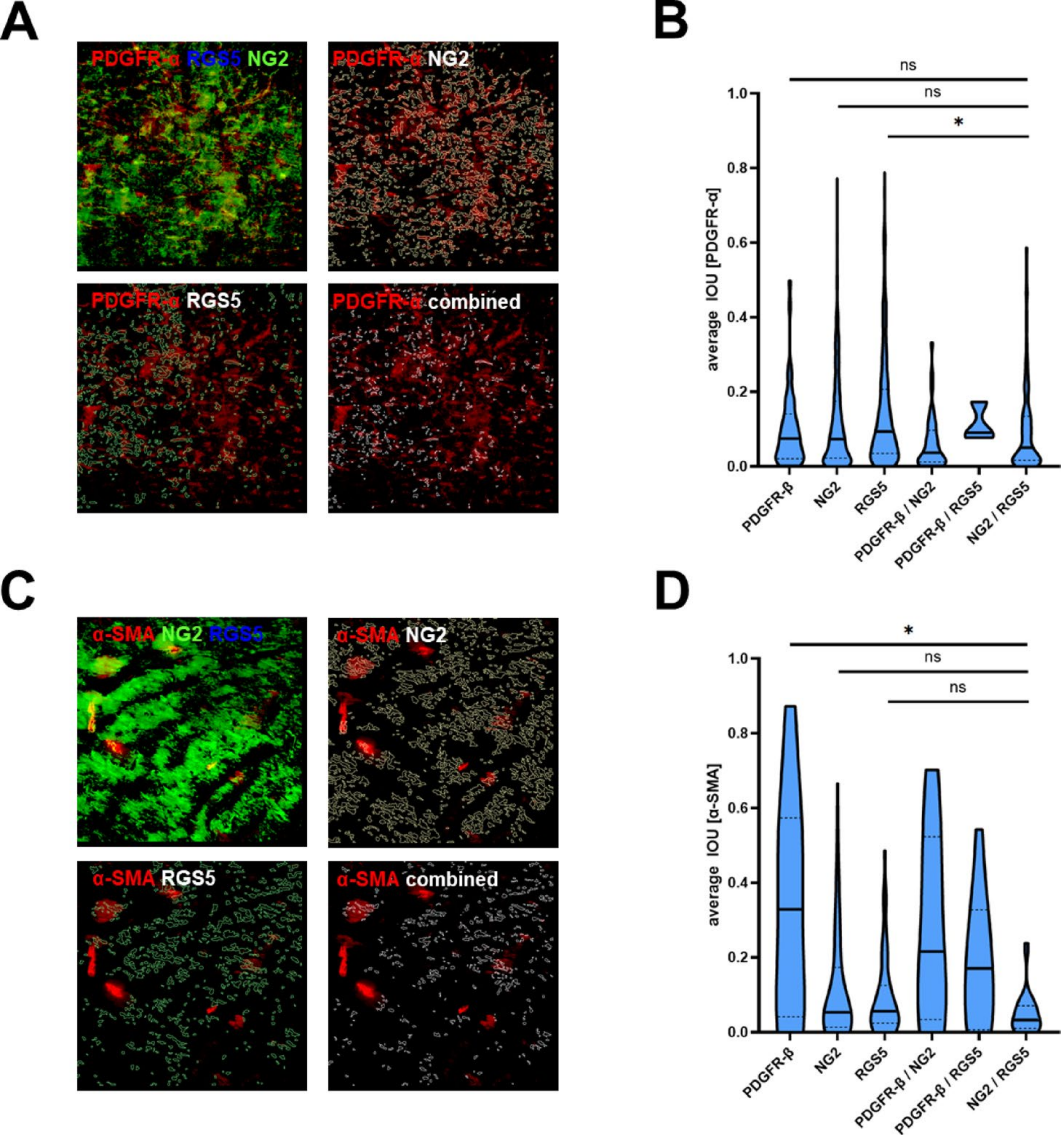
(**A**) the example picture of a triple staining shows PDGFR-α stained in red throughout with an overlay of NG2 outlines (upper right panel), RGS5 outlines (bottom left) and an overlay of the double positive outlines (lower right panel). (**B**) Average IOUs of different marker combinations against PDGFR-α shows a diminished overlap with the combination of NG2/RGS5. (**C**) the example picture of a triple staining shows α-SMA stained in red throughout with an overlay of NG2 outlines (upper right panel), RGS5 outlines (bottom left) and an overlay of the double positive outlines (lower right panel) (**D**) Average IOUs of different marker combinations against α-SMA shows a diminished overlap with the combination of NG2/RGS5 (* *p* < 0.05 / ** *p* < 0.001).

Pericytes are notoriously difficult to identify in tissue sections, since most common pericyte markers stain additional cell types, such as vascular smooth muscle cells and fibroblasts. In order to more precisely identify pericytes in tissue sections, we employed a double staining approach using the well-established pericyte markers NG2 and PDGFR-β as well as a novel pericyte marker RGS5^36^. Using double stainings for all possible combinations of those three markers, we generated images, which were then analyzed by CAPPER. We identified pericytes as those cells which show double stainings for both markers and compared overlap to unintended stainings of vascular smooth muscle cells and fibroblasts. Using CAPPER we could show, that staining with PDGFR-β alone leads to substantial staining of fibroblasts (PDGFR-α, Fig. 4 A + B) and vascular smooth muscle cells (α-SMA, Fig. 4 C + D). While the effect was less pronounced in NG2-positive cells and RGS5-positive cells, there still was a substantial overlap of these stainings with undesired fibroblasts and vSMCs. Interestingly, the combination of RGS5 and NG2 displayed a good staining for pericytes while limiting aberrant fibroblast and vSMC stainings. To further elaborate on this finding, we isolated pericytes with single or double marker combinations and performed qPCR to determine the expression of fibroblast and vSMC markers in these cells. Here also, the combination of RGS5 and NG2 was most capable of eliminating co-isolation of fibroblasts and vSMCs (Supplementary Fig. 3 A + B). These data indicate that a double staining with RGS5 and NG2 most accurately identifies pericytes, which are notoriously elusive, aiding in the further examination of a cell type crucial in the development of most vascular disorders.

## Discussion

Quantitative assessment of vascular architecture is fundamental to understanding tissue physiology and remodeling in health and disease. Yet reliable quantification of capillary density and pericyte coverage from histological images remains challenging due to staining variability, tissue heterogeneity, and the complex spatial relationship between endothelial and mural cells. In this study, we introduce an automated, algorithm-based approach named CAPPER (Capillary / Pericyte Quantification Tool) that standardizes capillary and pericyte analysis across organs and species. CAPPER integrates adaptive thresholding, morphological filtering, and domain-specific knowledge, yielding reproducible, examiner-independent measurements of both capillary density and pericyte coverage in immunohistochemical tissue sections.

CAPPER accurately reproduced known organ-specific differences in capillary density, with the heart showing the highest density, followed by kidney, skeletal muscle and lastly brain. These results are consistent with prior histological studies reporting comparable values^10,13–15,37^. Furthermore, CAPPER performs as expected in a plethora of conditions in mice and pigs, from exercise, diabetes, hypercholesterolemia, pressure overload, sepsis as well as following rAAV based gene therapy approaches. Importantly, CAPPER achieves this quantification in a fully automated, high-throughput manner, analyzing entire histological images within seconds. The introduction of an automated analysis of pericyte coverage, defined as the NG2-positive area fraction within CD31-positive capillary contours, represents a novel feature compared to existing histological analysis tools. Unlike absolute pericyte numbers, which lack information on endothelial association, this parameter integrates pericyte abundance with their spatial relationship to the endothelium, thereby capturing a critical determinant of vessel stability and vascular barrier integrity. The observed pattern of pericyte coverage, highest in the brain and lowest in the kidney, aligns with previous histological analyses^16,38^. CAPPER also revealed distinct heterogeneity in pericyte coverage among organs, suggesting tissue-specific differences in vascular organization and mural-endothelial coupling.

Automated vascular analysis has advanced considerably with the emergence of digital microscopy and computational image processing, and a range of dedicated workflows for capillary and pericyte quantification have been proposed. These include the QuPath-based whole-brain pipeline of Courtney et al.^4^, stereological and two-photon microscopy-based approaches in the central nervous system^5^, recent transition-zone and vascular-leakage-focused pericyte workflows in the retina^6,7^, and more general vascular analysis tools for the brain^8^. Beyond these, additional studies have quantified endothelial cell density in specific disease contexts, for example in breast cancer tissue using endothelial markers^39,40^. Despite this progress, most existing methods remain fundamentally limited by their focus on a single tissue type, reliance on transgenic reporter lines, or restriction to capillary density measurements without integrating per-capillary pericyte coverage. Endothelial cells and pericytes are frequently quantified independently, without assessing their spatial colocalization or functional interaction, and where per-capillary pericyte coverage is incorporated as a more biologically meaningful parameter, the analysis is typically confined to a single tissue (e.g. brain^8^ or retina^6^ or to in vitro and ex vivo systems such as cell or tissue cultures^41^. Consequently, a broadly applicable and robust pipeline that combines capillary density, pericyte coverage, and marker specificity across tissues, species, and imaging platforms has remained lacking.

CAPPER addresses this bottleneck by combining local adaptive thresholding, based on the maximum-curvature method described by Klauschen et al., with dual-channel analysis of endothelial and pericyte markers in static tissue sections ^9^. Tile-wise image processing compensates for staining heterogeneity and uneven illumination, enabling robust quantification across organs, staining protocols, and imaging systems. In contrast to previous approaches, the workflow performs per-capillary analysis of pericyte coverage by applying CD31-based masks to pericyte marker signals, thereby minimizing false-positive inclusion of surrounding mesenchymal or stromal cells. Importantly, thresholds for marker channels are derived dynamically from image-specific intensity histograms, and key segmentation parameters, including capillary diameter, are adaptively determined within biologically plausible ranges. This design ensures robustness against variability in staining intensity, antibody performance, and imaging conditions, while requiring only minimal parameter tuning when adapting the pipeline to new tissues or experimental setups. In practice, porting CAPPER to a new type of tissue, species or microscope setup typically requires adjustment of five parameters at the most, which are exposed in the software configuration: the tile size (to compensate for uneven staining and illumination across whole-section scans), the slope-recovery threshold of the histogram-based auto-thresholding (to account for differences in signal-to-background ratio), the Gaussian adaptive-thresholding kernel size (in our pipeline ~ 20 μm for mouse and ~ 60 μm for pig tissue reflecting an increased inter-capillary distance), the kernel sizes used in the morphological operations (larger closing reduces small spurious detections, while larger opening separates overlapping endothelial signals), and the capillary diameter gating range. We recommend that users verify automated segmentation against a small, manually annotated subset when porting CAPPER to a new staining protocol or imaging platform, particularly when antibody lots or imaging hardware differ substantially from those used here.

A central challenge in pericyte analysis is their molecular heterogeneity and the limited specificity of commonly used markers such as NG2 or PDGFR-β, which partially overlap with fibroblasts and vascular smooth muscle cells^16^. Consequently, the primary CAPPER workflow shown in Figs. 2 and 3 relies on CD31/NG2-based segmentation and does not explicitly discriminate capillary pericytes from vascular smooth muscle cells or other NG2-expressing mural cells at the segmentation step. This is a shared limitation of virtually all single-marker-based automated workflows and reflects the absence of a universally specific pericyte marker^16^. Importantly, however, the architecture of the pipeline is not restricted to the CD31/NG2 marker pair, and we address this limitation directly in the second part of the study (Fig. 4, Supplementary Fig. 3). By agnostically evaluating multiple pericyte markers and combinations, we identified the combination of RGS5 and NG2 as optimal for distinguishing pericytes from other mural or stromal cells. Both immunofluorescence colocalization and transcriptional validation confirmed the specificity of this combination. Previous reports have highlighted the value of RGS5 as a pericyte-enriched marker in angiogenic and pathological contexts^36,42^, and its integration into our algorithm further increases detection specificity. Future applications of CAPPER can therefore move beyond the limitations of single-marker segmentation by combining endothelial markers with multi-marker pericyte panels. Here, RGS5 and NG2 have shown to be most useful in combination with the same automated pipeline, enabling cleaner separation of pericytes from vascular smooth muscle cells and fibroblasts in routine histological sections. This flexible multi-marker capability represents a unique strength of the present framework and supports empirical optimization of staining strategies across tissues, disease models, and laboratories.

In summary, we present an algorithmic framework that unifies automated quantification of capillary density and pericyte coverage from standard immunohistochemical images. The main advantage of this pipeline lies in its balance between automation, transparency, and adaptability, and it achieves computational efficiency, high reproducibility, and compatibility with standard microscopic imaging setups, making it suitable for both basic research and translational pathology. CAPPER complements existing in vivo and histological approaches and enables systematic evaluation of vascular structures and mural cell interactions across health and disease. Thus, CAPPER offers a practical and scalable method permitting use of existing microscopic tools to further advance vascular biology and tissue pathology research.

## Materials & methods

### Animals

Our study examined male mice because male animals exhibited less variability in phenotype. It is unknown whether the findings are relevant for female mice. All animal experiments were approved by the appropriate governmental body (Bavarian Animal Care and Use Committee) with the following file numbers: ROB-55.2-2532.Vet_ 02-21-28 / ROB-55.2-2532.Vet_02-19-17 / ROB-55.2-2532.Vet_02-19-1 / ROB-55.2-2532.Vet_02-16-127 / ROB-55.2-2532.Vet_02-21-200 / ROB-55.2-2532.Vet_02-19-180. All experiments conformed to the Guide for the Care and Use of Laboratory Animals published by the NIH (NIH publication no. 85 − 23, revised 1996).

### Animal models

#### Murine model of ischemia/reperfusion

Ischemia was performed as previously described^43,44^. In short, mice were anesthetized with 2% isoflurane in combination with an intraperitoneal injection of fentanyl (0.05 mg/kg), midazolam (5.0 mg/kg), and medetomidine (0.5 mg/kg). After sedation, mice were intubated orally and ventilated using a MiniVent system (MiniVent Ventilator model nr. 845, Harvard Apparatus) with a volume of 150 µl at a respiration rate of 200 / min. After lateral thoracotomy, the left anterior descending artery was ligated with an 8 − 0 prolene suture leading to an ischemic area at the left ventricular apex. After 60 min the suture was removed and recurring blood flow was observed visually. Following the operation, analgesia was continued for 3 days post-surgery twice per day with buprenorphine (0.1 mg/kg). Organs were harvested after 30 days. Mice were obtained from Charles River Laboratories and euthanized under isoflurane anesthesia, followed by cervical dislocation.

#### Murine model of transverse aortic constriction (TAC)

Adult mice were first anesthetized using a combination of midazolam (5.0 mg/kg), medetomidine (0.5 mg/kg) and fentanyl (0.05 mg/kg), which was injected intraperitoneally. Upon sedation, mice were intubated and ventilated (MiniVent Ventilator model nr. 845, Harvard Apparatus) with a volume of 200 µl at a respiration rate of 200 / min and placed in a supine position. buprenorphine (0.05 mg/kg) was injected prior to the surgery. A partial sternotomy was performed at the second intercostal space and the aortic arch was uncovered. To induce pressure overload, the aortic arch was banded between the innominate artery and left common carotid artery with a 27-gauge needle using a 5 − 0 silk suture^45,46^. The sternum was readapted and the skin sutured. Anesthesia was antagonized with a subcutaneous injection of flumazenil (0.5 mg/kg) and atipamezole (2.5 mg/kg). Post-operative analgesia was ensured via buprenorphine (0.05 mg/kg) three times daily. Organs were harvested after 8 weeks. Mice were obtained from Charles River Laboratories and euthanized under isoflurane anesthesia, followed by cervical dislocation.

#### Murine model of systemic inflammation

To induce a sepsis-like phenotype, adult mice were injected with lipopolysaccharides from E. coli (20 mg/kg, serotype O55:B5, L2880, Sigma-Aldrich). To ensure analgesia throughout the experiment, mice were injected with buprenorphine (0.05 mg/kg) at sepsis induction and every 12 h. Sepsis severity was assessed at 12, 24, 36, 48, 72 and 96 h after onset of systemic inflammation using a scoring system. Mice were obtained from Charles River Laboratories and euthanized under isoflurane anesthesia, followed by cervical dislocation.

#### Murine exercise model

To investigate the effect of physically active behavior vs. sedentary behavior mice either had access to a running wheel or were housed without a wheel for 6 weeks. Mice were obtained from Charles River Laboratories and euthanized under isoflurane anesthesia, followed by cervical dislocation.

#### Porcine models of hypercholesterolemia and diabetes mellitus

To induce hypercholesterolemia in pigs, wild-type Landrace pigs were fed a high-fat diet as described previously^23^. These animals demonstrate elevated cholesterol as well as triglyceride blood levels without demonstrable increase in circulating glucose or insulin levels.

To mimic a diabetic state, transgenic INS^C94Y^ pig lines were used^22,47^. This mutation disrupts one of two disulfide bonds between the insulins A and B chain, resulting in a misfolding of the protein. This leads to impaired insulin secretion, endoplasmic reticulum (ER) stress and apoptosis of the pancreatic beta cells. Fasting glucose levels in these animals are increased to 300–400 mg/dl after birth. All pigs were sourced from the Center for Innovative Medical Models (CiMM) of the veterinary faculty, LMU Munich and were euthanized by intracardiac injection of saturated potassium chloride under full propofol/fentanyl anesthesia.

#### Porcine model of chronic hindlimb ischemia

As a model of porcine hindlimb ischemia, we established the percutaneous implantation of a modified Bentley reduction stent (90% stenosis) into the superficial femoral artery, where it occluded towards the end of the implant procedure. To treat the animals with either rAAV.MRTF-A or rAAV.GFP as a control, a microcatheter for vector injection was advanced through the reduction stent at the end of the procedure to ensure distribution of the vector throughout the ischemic limb. 5 × 10^14^ viral genomes were injected per animal. Pigs were euthanized 5 weeks after stent placement by intracardiac injection of saturated potassium chloride under full propofol/fentanyl anesthesia. Immediately after, tissues were harvested and preserved for analysis. All pigs were sourced from the Center for Innovative Medical Models (CiMM) of the veterinary faculty, LMU Munich.

### Histological analysis

Hearts, brains, kidneys, and peripheral muscles were harvested upon termination of the experiment. The harvested organs were fixed in 4% PFA and embedded in Tissue-Tek OCT compound (Sakura Finetek, Staufen, Germany), frozen on dry ice and stored at -80 °C until further use. Cryosections of 10 μm thickness were cut on a cryotome. Samples were thawed, permeabilized with 0.1% Triton X-100 in PBS and blocked with 5% bovine serum albumin / 0.1% Triton X-100 in PBS for 1 h. Streptavidin-Biotin blocking was performed in the respective samples using Invitrogen™ ReadyProbes™ Streptavidin/Biotin Blocking Solution (Thermo Fisher Scientific, R37628). Where host species of primary antibodies in a sample would collide, respective antibodies were labeled with fluorophores using the Invitrogen™ Labeling Kits (Thermo Fisher Scientific, A-88062 and A-88068) after purification with Pierce™ Antibody Clean-up Kit (Thermo Fisher Scientific, 44600). Primary and secondary antibodies were incubated in blocking solution. Samples were embedded in Vecta Shield Mounting Medium. The following antibodies were used: CD31 (ACRIS, BM4086), NG2 (Millipore, AB5320B), RGS5 (Proteintech, 11590-1-AP), PDGFR-α (Biotechne, AF1062), PDGFR-β (Biotechne, BAF1042), α-SMA (Sigma, C6198), CD31 (Bio Rad, MCA1747), NG2 (Prestige, HPA002951). All secondary antibodies were purchased from Thermo Fisher (Thermo Fisher Scientific, Waltham, MA, USA): anti-rabbit A488 (A-11008 and 21206), anti-rabbit A546 (A-11035 and 10040), anti-rabbit A647 (A-21245 and A-31573), anti-mouse A488 (A-11001), anti-mouse A546 (A-11003), anti-rat A488 (A-11006), anti-rat A647 (A-21247 and A-78947), anti-goat A488 (A-11055), anti-goat A546 (A-11056), anti-goat A647 (A-21447), Streptavidin A546 (S-11225) and Streptavidin A647 (S-21374).

Images were acquired on a Leica SP8 Confocal Microscope (Leica Microsystems, Wetzlar, Germany) and a Leica DMi8 using a 40X magnification.

### Algorithm

The algorithm was developed in Python version 3.12.12. The implementation of core image processing operations such as adaptive thresholding and morphological transformations was provided by OpenCV^48^ via the wrapper package opencv-python version 4.12.0. Further mathematical operations on images were done using NumPy version 2.0.2^49^ and SciPy version 1.16.3^50^. The generation of data sheets was performed using xlsxwriter version 3.2.9. Data visualization in the form of plots and images was generated with Matplotlib version 3.10.0^51^. The algorithm has been tested and executed on Google’s cloud computing platform Colab using a CPU (AMD EPYC 7B12) runtime with extended RAM (51.0 GB).

### Algorithm for mouse tissue

To establish a reproducible workflow for automated vascular analysis, we implemented an algorithm that combines adaptive thresholding with morphological filtering to process large-scale immunohistochemical images. The pipeline begins with co-detection of PECAM-1 (CD31) to identify endothelial cells and NG2 to label pericytes. High-resolution images were exported at 16-bit color depth and separated into CD31 and NG2 channels. To account for uneven staining intensity, illumination variability, and imaging artifacts, images were divided into tiles of 500 × 500 pixels for local processing.

Automatic thresholding was performed on each channel using a maximum curvature estimation approach, following the protocol of Klauschen et al.^9^. Histograms of pixel intensities were generated and filtered with a median filter (filter window size: 255). Peaks representing tissue background and holes in the specimen were identified, and thresholds were defined based on gradient analysis of the histogram curve, selecting the point where the slope of the histogram recovered to 5% of its maximum after the tissue background peak for the CD31 signal and respectively 70% for the NG2 signal. The resulting thresholded images were converted to 8-bit for further analysis.

Endothelial structures were identified by detecting contours of the CD31 signal, preceded by Gaussian blurring and Gaussian adaptive thresholding with a kernel block size equivalent to 20 μm. Morphological closing, using a circular binary kernel with a diameter of the pixel equivalent of 1.5 μm, ensured coherent capillary shapes, while two consecutive opening operations, with 2 × 6 pixel and 6 × 2 pixel elliptical binary kernels respectively, removed spurious connections in horizontal and vertical orientation. Now, objects were detected by a contour finding algorithm. The area of detected objects was then analyzed to further filter out false signals, which are either too large or too small to come from capillaries. Here an adaptive minimum size filter was derived from the histogram of contour diameters by focusing on its bimodal nature. Two dominant peaks were identified, corresponding to spurious signals and true capillaries, and were required to be separated by at least a diameter margin of 0.5 μm. The most prominent valley between these two peaks was defined as the minimum diameter threshold, providing a conservative cut off. This minimum diameter was limited to the range between 3.5 μm and 5 μm. Any automatically detected value outside these bounds would be clipped to the respective end. In the special case of brain tissue, a manually fixed lower diameter limit was set at 8 μm due to the scarcity of cross-sectionally cut capillaries and a high amount of background noise. The upper diameter limit for capillaries was always set to 12.0 μm. For each identified capillary, the corresponding NG2 signal was assessed.

To quantify pericyte coverage, each CD31-positive contour was used to crop a region in the auto-thresholded NG2 channel with a pixel margin equivalent to 1.5 μm. A mask generated from the CD31 outline was applied to ensure that only NG2 signals within the capillary boundary were analyzed. The pericyte area was thresholded adaptively (Gaussian adaptive threshold) with a kernel block size half the size of the cropped region. Finally, the pericyte coverage was calculated as the ratio of NG2-positive signal to CD31-positive signal within each contour.

### Adaptation to pig tissue

We employed a mostly identical program to analyze images of porcine tissue with small alterations that account for the differences in staining quality and signal to background ratio. Firstly, the tile dimensions have been enlarged to 3000 × 3000 pixels to extract a more robust average of the background pixel values. In the automatic thresholding procedure, the original histogram curves were additionally smoothed through a convolution operation. The slope recovery threshold has been set to 70% for both the CD31 and the NG2 signal. Gaussian adaptive thresholding to detect the CD31 signal was performed with a kernel block size equivalent to 60 μm. The morphological closing kernel dimensions were enlarged to the pixel equivalent of 5 μm. Lastly the adaptive size filtering was discarded for a fixed gating set to the ranges 2–20 μm in diameter for heart, kidney, skeletal muscle and 10–20 μm respectively for brain tissue.

### Evaluation of pericyte markers

High-resolution images were processed in tiles of 500 × 500 pixels. Each tile was subjected to automated thresholding analogous to the procedure applied for mouse tissue samples. Cell structures were identified using the same approach as for CD31 signals in mouse tissue. Binary masks were generated for each staining channel, and pixel-level overlap between signals was computed to identify regions of co-expression. To evaluate the overlap between pericyte- (PDGFR-β, NG2, RGS5) and non-pericyte-markers (ɑ-SMA, PDGFR-ɑ), the intersection over union (IOU) was calculated pairwise between the pericyte and non-pericyte-signals. Given segmented fluorescent signals A & B the IOU is defined as the number of pixels within the bounds of both signals simultaneously (intersection) divided by the number of those within the bounds of one or the other (union):

$$IOU=\frac{{A \cap B}}{{A \cup B}}$$

### Statistical analysis

All statistical analyses were performed on the tile-level quantitative read-outs (capillary density, pericyte coverage, IOU) generated by CAPPER, using GraphPad Prism (version 10). Data are presented as mean ± standard error of the mean (SEM). Normality of data distribution was assessed using the Shapiro-Wilk test, and non-parametric tests were applied whenever the assumption of normality was violated.

Comparisons between two independent groups (exercise vs. sedentary in Fig. 3A–C; rAAV.GFP vs. rAAV.MRTF-A in Fig. 3J–L) were performed using an unpaired two-tailed Welch’s t-test, or a Mann-Whitney U-test in case of non-normally distributed data. For comparisons between more than two groups across the full set of pairwise contrasts (Fig. 2B, C, E, F), a one-way ANOVA followed by Šídák’s multiple-comparison test was applied, with Kruskal-Wallis followed by Dunn’s test used as a non-parametric alternative.

For comparisons of experimental groups against a common reference group (hypercholesterolemic and diabetic pigs vs. wild-type in Fig. 3D–F; I/R, TAC, and sepsis vs. healthy control in Fig. 3G–I and Supplementary Fig. 2A, B), a one-way ANOVA followed by Dunnett’s multiple-comparison test against the control group was used; in case of non-normally distributed data, Kruskal-Wallis followed by Dunn’s multiple-comparison test against the control group was applied as a non-parametric alternative, and pairwise group-vs.-control comparisons were additionally verified with Mann-Whitney U tests with Bonferroni correction across the relevant set of contrasts. For the pairwise comparisons between disease groups in Fig. 3G–I and Supplementary Fig. 2A, B, Šídák’s correction across the three disease-group contrasts was applied to the parametric analysis, with Dunn’s correction used in the non-parametric setting.

For the marker-combination analyses in Fig. 4B, D, pericyte-marker combinations were compared against the RGS5 + NG2 reference using Mann-Whitney U tests with Bonferroni correction for a pre-specified set of biologically relevant pairwise comparisons (two comparisons in Fig. 4B, three in Fig. 4D); non-parametric testing was used because IOU values are bounded between 0 and 1 and were non-normally distributed. The qPCR-based marker-combination comparisons in Supplementary Fig. 3A, B were analysed by one-way ANOVA followed by Dunnett’s test against the RGS5 + NG2 reference.

Because tiles within the same image and animal are not fully statistically independent, the primary tile-level analysis was complemented by a nested linear mixed-effects model with “animal” as a random effect in a sensitivity analysis, which yielded qualitatively identical group-level conclusions. A two-sided p-value of < 0.05 was considered statistically significant; in figures, * denotes *p* < 0.05, ** *p* < 0.001, and # *p* < 0.05 vs. control where indicated. Detailed information on the number of animals, images, and analysis tiles per experimental group is provided in Supplementary Table 1.

## Supplementary Information

Below is the link to the electronic supplementary material.

Supplementary Material 1

## Data Availability

The CAPPER source code, together with installation instructions and a user guide including an example workflow, will be made publicly available under an open-source license at https://github.com/ariogato/capper. Raw image data and representative example tiles supporting the findings of this study are available from the corresponding author upon reasonable request.
