# Supplementary material for: Algorithm-based quantification of tissue vascularization in immunohistochemical stainings of tissue sections
Source: Sci Rep. 2026 Jun 8;16:17700. doi: 10.1038/s41598-026-56867-x (PMC13246862; doi:10.1038/s41598-026-56867-x)
Supplement: Supplementary file 1 — Supplementary Material 1 [file 41598_2026_56867_MOESM1_ESM.pdf]

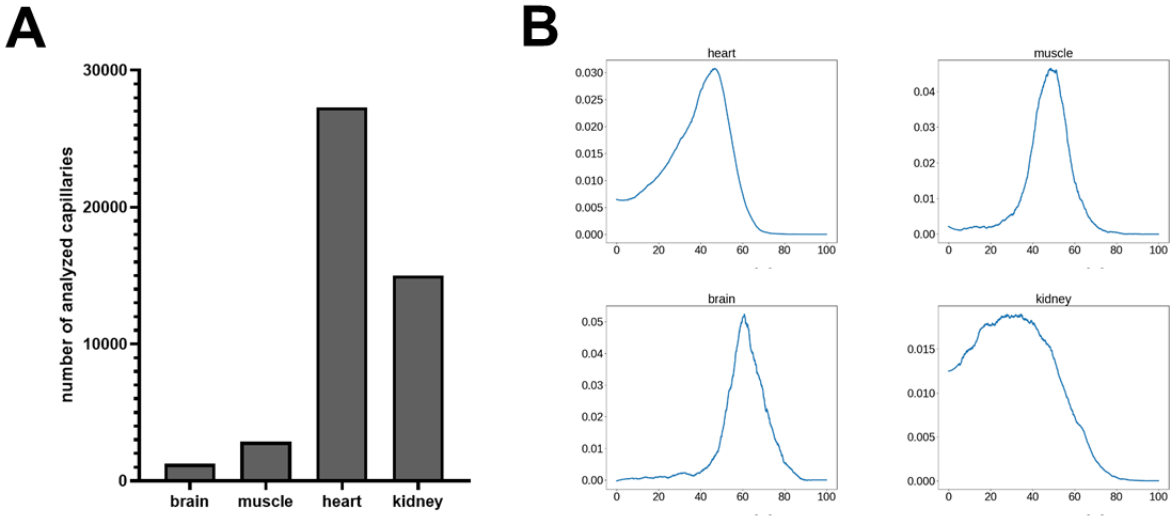

Supp. Fig. 1 A Due to differences in capillary density throughout the organs, different amounts of capillaries were investigated for pericyte coverage **B** Histograms of pericyte coverage in brain, muscle, heart and kidney demonstrate a high variability in coverage in kidneys with a rather narrow distribution in the brain and muscle.

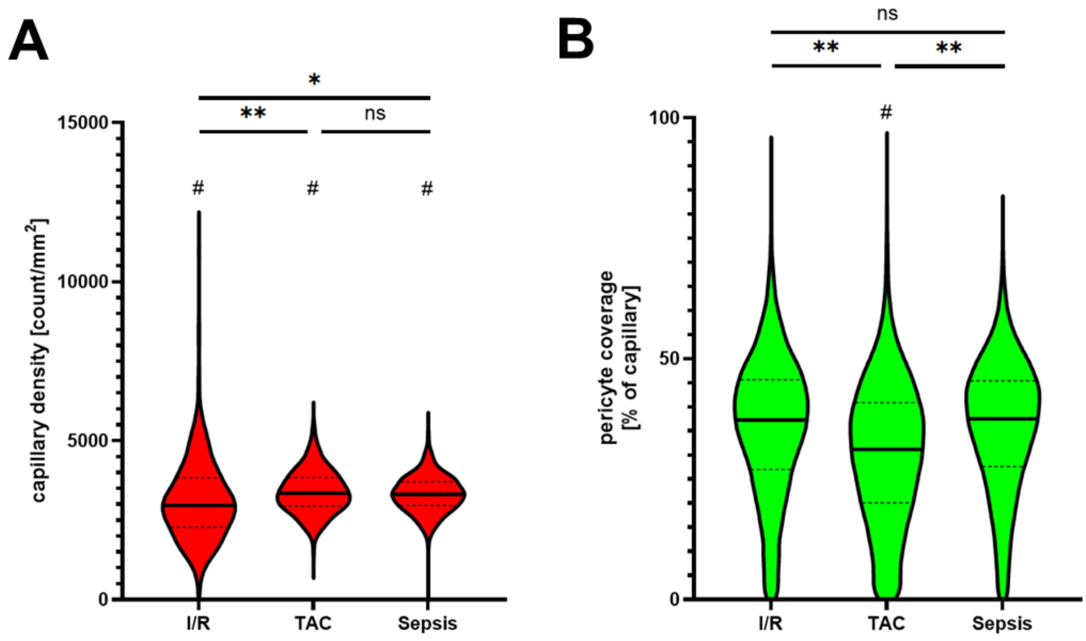

Supp. Fig. 2 A Capillary density (in counts/mm^2) and **B** pericyte coverage (as a coverage percentage of capillaries) of murine hearts in different disease states shows a decrease in capillary density in all states, most prominently in the ischemic myocardium with a less pronounced decrease in pericyte coverage, while the highest loss in pericyte coverage can be seen during TAC (* p<0.05 / ** p<0.001 / # p<0.001 vs. Ctrl.)

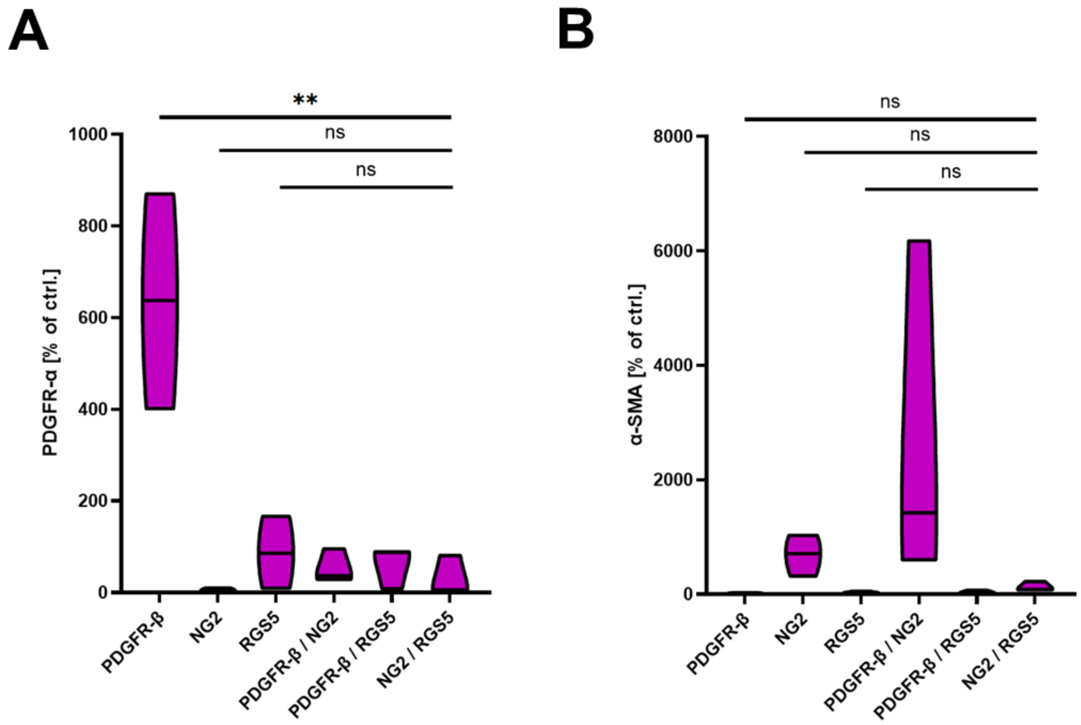

Supp. Fig. 3 qPCR analysis of **A** PDGFR-α expression and **B** α-SMA expression in cells FACS-sorted after staining for the indicated pericyte markers shows a low degree of off-target marker expression in NG2/RGS5 doulbe positive cells (** $p<0.001$)

	group	# animals	# images	# tiles
mice	brain	3	9	1336
	kidney	3	9	1089
	muscle	3	9	1089
	heart	3	12	1452
	I/R	3	23	1359
	sepsis	3	57	1181
	TAC	3	69	1168
	exercise quad	3	27	6629
pigs	brain	3	3	33
	heart	3	3	19
	kidney	3	3	24
	muscle	3	3	9
	contralateral WT	7	7	31
	ischemia + MRTFA WT	3	6	44
	ischemia WT	3	5	32
	contralateral CHOL	5	5	37
	contralateral DM	5	5	29

Supp. Table 1 Number of animals per group, images per group and analyzed tiles per group
